# Medico-Chirurgical Transactions

**Published:** 1817-02

**Authors:** 


### *Medico-Chirurgical Transactions, Vol. VII. Part I.*

( Continued from our last Number, page 32. )

ART. 7. *Aneurism of the Femoral Artery treated by Ligature of the external Iliac:* by CHARLES COLLIER, Esq. Surgeon to the Forces.

JOHN MORRISY, ætat. 24, had been wounded by a musket ball in June, which entered the thigh three inches below Poupart's ligament, where the cicatrix was still visible on the 24th of August. Tumour, now three inches in length by two in breadth, extending to within an inch of the above-mentioned ligament. Pulsation powerful, thrilling, and resisting. Skin and temperature of the limb natural; constitution vigorous. Operation was performed on the 28th of August, thus:

"I made a semicircular incision, which began three-fourths of an inch on the inner ring, had its base on Poupart's ligament, and terminated about one inch and a half from the anterior superior spinous process of the ileum, horizontally with the commencement. The fascia of the external oblique, after being exposed, was detached from Poupart's ligament, to the same extent, and in the same direction; on turning it up, the lower edge of the internal oblique, and the beginning of the cremaster muscles, with the spermatic cord passing through the inner ring, were distinctly seen. Some fibres of the internal oblique, having their origin from the ligament, were then divided, in order to give me room. The cord was held aside with the flap formed by the incision, while I enlarged the inner ring with the handle of the knife, and detached the peritoneum with my finger, so as to enable me to feel the artery, which I found covered by, and closely connected to small glands, the largest of which I removed. Having separated the artery, I passed a director under it, and the gentlemen present being satisfied that it alone was raised, I passed a probe, armed with a ligature, along the groove, and secured it; the pulsation in the aneurismal tumour instantly ceased, the edges of the wound were aproximated by one suture. Two small arteries sprang during the operation, but there was no blood lost deserving the least consideration." p. 139.

In three or four hours after the operation the limb lost its temperature, and the patient was anxious. In seven hours the anxiety had increased, and the pain was excessive. Some discoloured patches were discovered on the calf of the leg. He was very restless during the night; the pulse intermitting. 29. Omnia exacerbata. 30th. The wound was dressed, and appeared irritable; pulse full and hard; skin hot. Was bled to sixteen ounces; the blood cupped. Two sphacelated spots near the ankle joint. Was bled again on the 31st, which gave some relief. He died on the 1st of September.

*Post mortem.* On opening the abdomen, there appeared a general flush of the intestines, but nothing else particular. The whole limb gangrenous.

Such a termination was hardly to be expected in this case.

Art. 8. *On the Medical Properties of the Pyrola Umbellata, and Arbutus Uva Ursi.* By Professor Barton, of Philadelphia.

Our readers will recollect the account we gave of Dr. Somerville's paper on the Pyrola umbellata in the tenth volume of the *New Medical Journal.* The plant has been described by Professor Barton in his "Collections, &c." and the present paper contains some additional observations. He conceives, that a considerable analogy subsists between the pyrola and the uva ursi, since it occasionally renders the urine black. The diuretic effects however, of the pyrola, have not been much noticed by the American practitioners. The North American Indians employ it in rheumatism and fever, in the form of a strong decoction of the whole plant; which, when given in large quantities, excites a copious perspiration. Many of the white inhabitants follow the practice of the Indians. Professor Barton believes, that the plant possesses strong anti-lithic powers; not less so than the uva ursi, of which our author entertains a very high opinion. Notwithstanding the declamations concerning the paucity of diseases among the American Indians, it appears, that they are by no means exempt from many of those grievous maladies which so often embitter the days of civilized man. Calculous complaints, for example, are very prevalent among them. There is no other information in the Professor's paper, excepting a hint respecting "the happy effects of blood-letting, of mild cathartics, and of a vegetable diet in many cases of dropsy." 148.

Art. 9. *Ossification of the Larynx preventing Deglutition.* By Dr. F. Travers, of Newark.

This was a woman, ætat. 50, who had enjoyed good health till the spring of 1815, when she began to experience dysphagia, attended by an enlargement of one of the tonsils. In October, the tonsil had increased to the size of a walnut; was firm in structure; and protruded considerably over the entrance to the pharynx. Mr. Calton removed the gland, by passing a ligature through its centre, and then cutting it out with a small scalpel. The dysphagia continued, and upon passing the index finger of the right hand as far as it could reach down the œsophagus, a projection was distinctly felt filling up the whole of the canal, except at its left and posterior part, where only a small dilatation could be perceived. It could not be dilated farther, and the patient died. The cricoid and arytenoid cartilages were much increased in size, and completely ossified. They caused the obstruction by pressure on the œsophagus, which, in itself, was natural.

Art. 10, Is a *well written* paper in latin, by Dr. Somerville, on the peculiar structure of the female organs of generation in the Hottentot. It is utterly devoid, however, of any practical utility ; nor do we see any thing added to our stock of information on the subject. The following passage contains the whole.

"Ex interiore parte rimæ pudendi, substantia laxa, pendula ac sæpe rugosa descendit, quæ curiosius explorata duplex esse reperitur, ex nymphis productis constans, adeo arcte inter se cohærentibus ut simplex esse videatur. *Nymphæ in quibusdam extra marginem labiorum quinque uncias descendunt.*"

Dr. S. can offer no natural cause for this peculiarity of structure; and his conjectures respecting the origin of the Caffries we need not transcribe or notice, since they have nothing to do with medicine or any of its auxiliaries.

Art. 11. *Removal of the Head of the Os Humeri with Part of the Humerus.* By W. R. Morel, Esq. Surgeon to the Forces, &c.

The preservation of a limb is at all times desirable, and the attempt is infinitely more praise-worthy than the most dexterous operation.

Thomas Ellard, a private in the hussars, received a shot through the left shoulder, in the battle of Waterloo. The ball had passed through the head of the bone about an inch and a half below the scapular extremity of the clavicle. When received into the York hospital in September, 1815, there were three wounds; two from the entrance and exit of the ball, and one from the bursting of an abscess. Through the latter, some fragments of bone had been extracted; and when a probe was introduced, shattered pieces of bone could be felt in every direction. The irritation and discharge were making inroads on the man's constitution, and nature appeared unable to remove the offending cause.

*Operation.* The subclavian artery being compressed, the deltoid muscle was raised as far as for amputation at the joint. In this, some difficulty was experienced from the condensation of cellular membrane. The flap being raised, the head of the bone was dissected out; the saw was applied to the shaft of the humerus where the tendon of the pectoralis is inserted, and the anterior half of the bone was removed. This gave an opportunity for examining the remaining half, which was thrown back under the edge of the cup of the scapula. To remove this, it became necessary to separate the capsular ligament from its connection with the neck of the bone and contiguous muscles. The remaining portion of bone was removed by the saw, and the flap was adjusted and preserved in its place by adhesive straps and bandages. In the operation, two pounds of blood were lost, and two arteries tied. The recovery was tedious, and at one time the inflammation ran so high, that the event was very doubtful. Mr. Morel is rather too prolix in the detail of subsequent treatment, and in many places obscure, if not tautological; for instance, "pulse 100, and quick." p. 167. There was finally but the difference of an inch and a quarter in length between it and the other arm. There was little motion of the shoulder, but all the variety of motions of the fore-arm and hand were preserved.

**Art. 12.** *Influence of the Nervous System in regulating Animal Heat.* By Henry Earle, Esq.

Previously to Mr. Brodie's experiments, the animal temperature was generally supposed to be regulated by the functions of respiration; but this gentleman has proved, that the influence of the brain is not to be left out of the question: on the contrary, that it is essential to the production of animal heat. Mr. Earle has lately met with some cases of impaired nervous energy, which exhibited phænomena powerfully illustrative of the above position.

*Case* 1. Thomas Anderson, a mariner, had received a fall from the main yard into a boat alongside, in February, 1812, by which he was stunned and had his collar bone broken. This was followed by paralysis of the left arm, accompanied by violent pain when attempting to move the limb. This painful stage gradually abated, and the arm remained perfectly palsied and useless. Mr. E. suspected that the axillary plexus of nerves had been injured by the fracture. The pulse was unaffected; the temperature much reduced. Electricity raised the temperature of the hand four degrees; and in a few days he could feel a degree of warmth and tingling for some time after drawing the sparks. On a more accurate trial, ten days afterwards, electricity raised the temperature of the hand, six degrees; the arm three; and the axilla one degree. After some time sensation and motion began to return, though in a very irregular manner; one part of the arm possessing natural feeling, another being morbidly sensible, and another insensible. A blister was tried, and the denuded surface raised the thermometer three degrees. The progress of the case was slow, but it continued to advance till the patient went on board a ship as cook. The arm having once been immersed in a pail of warm grains, alarming blisters took place, and sloughing. From the circumstances of this case, Mr. Earle concludes, first, that a palsied limb is colder than natural, notwithstanding that the circulation is undisturbed. Second, that it is incapable of supporting any fixed temperature; and lastly, that it cannot, without injury, sustain a degree of warmth which would not be at all prejudicial to a healthy limb.

*Case* 2, Was a young girl who had long laboured under a neuralgic affection of the ulnar nerve, which resisted every mode of treatment but the knife. The nerve was laid bare by Mr. Earle, divided, and a part of it dissected out. The operation gave excruciating pain, but was attended with complete relief. The little finger and one half of the ring finger from that moment lost all sensation, and the integuments on the inside of the hand became perfectly void of feeling. The disease was eradicated. The little finger remained permanently colder than the others, and sloughed several times from atmospheric vicissitudes; the parts unsupplied by nervous energy being unable to support those variations of temperature which proved quite harmless to the rest of the hand.

In examining paralytic limbs, Mr. Earle has invariably found them colder than any other part of the body, unless artificially warmed. A friend of his lately examined twenty-five cases in the Bath Hospital, and found the paralysed limbs, in every instance, below the natural standard.

It is evident, that a perfect integrity of the nervous system is requisite to enable the body to resist the extraordinary variations of temperature to which it is so frequently exposed, and maintain a standard equilibrium. It is also evident, that important changes do take place during the transmission of blood through the lungs; and that such changes are essential to the support of nervous energy. Dr. Davy has proved by direct experiment, that the temperature of the arterial, is higher than that of the venous circulation; hence we have demonstrative evidence of the acquisition of heat during the pulmonary circulation. Defective supply of arterial blood, by depriving the brain of its accustomed stimulus, induces syncope and diminution of warmth in the body; while, on the other hand, a morbid increase of circulation, as in fever, deranges the nervous functions by over-excitement, and the temperature of the body is increased. Sudden determinations of blood to the nerves of any part, produce a local affection analogous to the more general derangement caused by sanguineous afflux to the brain. Thus, in many instances, amaurosis is remediable by copious bleeding. In no case thinks our author, is the effect of a local determination more marked than in *tic douloureux*. In every instance of this distressing malady which has fallen under his observation, during each paroxysm of pain, there was an evident increased flow of blood to the part, accompanied with an increase of heat more or less perceptible.

"In a very interesting case which I have lately attended, of affection of the nerves of the forehead and face, there was a well defined red line in the whole course of the supra-orbitary nerve, accompanied with so much heat, as rapidly to evaporate any cold washes which were applied. In another case, of a middle aged widow lady, who had been afflicted for years with *tic douloureux* of the inferior maxillary nerve, for the cure of which she had submitted to several operations, but who still at times suffered from pain in the deep seated temporal branches, the gustatory nerve, and all the branches supplying the masseter, pterygoid, and buccinator muscles; during the paroxysm, there was a violent pulsation in all the branches of the external carotid artery, which terminated generally in a most profuse flow of saliva, after which she experienced temporary relief.

"In all these affections, the local abstraction of blood, and the application of cold, are found most beneficial, and occasionally immediate ease is obtained, by forcibly compressing the part, and thus diminishing the influx of blood. A curious instance, in illustration of this, was lately mentioned to me. A blacksmith, who for many years had suffered from a violent pain in the nerves of the forehead whenever he exerted himself, accidentally found that he obtained ease by compressing the trunks of the temporal arteries; upon which, he contrived a strong spring, with a pad at each end, which he fixed on his head in such a manner, as to compress the temporal arteries. This spring he wore whenever he went to work, and was thus enabled to remain at the anvil all day without suffering.

"That there is an increased flow of blood to the part in these painful nervous affections, is not only shown by the redness and arterial action, but has been proved by an interesting dissection mentioned by Bichat, of a case of painful affection of the sciatic nerve, where the vessels of the neurilema were evidently enlarged in size, and increased in number, so as to be quite tortuous

"A singular instance, illustrating the effect of an inflammatory determination of blood to a nerve, occurred to me in the course of last year, and tends much to corroborate the preceding remarks.

"Maria Williams, a foundling, aged 32, who had been retained in the hospital in consequence of a deficiency of intellect, in February, 1814, wounded her arm with a fork, and punctured the external cutaneous nerve, about half way down the fore-arm. She experienced much pain soon after the accident, in the whole course of the nerve, and considerable inflammation took place in the neighbourhood of the wound. She was directed to keep the arm very quiet, and to apply evaporating washes. About three weeks after the accident, she had occasion to use the limb, when she was suddenly attacked with great pain and sense of burning in the seat of the original wound. Erysipelatous inflammation soon spread over the whole front of the fore-arm, which terminated in several large vesications, giving an appearance very similar to the disease termed Pemphigus. The heat of the arm was very great, and quickly dried the damp cloths which were applied. By perfect rest, and evaporating poultices with opium, she soon recovered from this attack ; but on attempting shortly after to use the arm, a recurrence of the same symptoms took place. The heat was so great this time, as to lead me to ascertain its extent with the thermometer, when I found that the mercury rose nearly three degrees higher when applied to the arm, than when placed under the tongue. At this time vesication had taken place in some parts, and probably the temperature was lower than it had been in the previous stage of inflammation. Subsequent to this, she experienced four several relapses, all apparently induced by inadvertently using the arm. The last attack was in September, and differed somewhat in its character from the former ; no vesications followed the inflammation, and the appearance bearing more analogy to urticaria than to pemphigus.

"The inflammation was always confined to the front of the fore-arm, and did not appear disposed to spread. The nerve, during the whole time, was acutely sensible when pressed. After September, she retained her arm constantly in a sling, for the space of three months, and has experienced no return of inflammation since." p. 190.

When a ligature is placed on the principal artery of a limb, the smaller vessels and capillaries are dilated, and a preternatural degree of heat soon succeeds. This, as Mr. E. justly observes, could not happen, if the temperature depended solely on the circulation, as the actual volume of blood is lessened. But by the distention of the smaller order of vessels and the capillaries; in short, of those vesels which immediately supply the nerves, the nervous system is over-exerted and more heat developed.

Upon the whole, this article is very interesting, and creditable to the talents of this rising young surgeon.

Art. 13. *Observations on the Treatment of Varicose Veins of the Legs.* By B. C. Brodie, Esq.

Every one knows the dangerous consequences which have resulted from ligature or even division of the saphena vien, for a varicose state of its branches in the leg. Mr. B. was led to think, that the same operations on the branches themselves would not be attended with similar bad effects. It was recommended by Celsus to employ the cautery in varices of the leg, or extirpate them altogether with the knife ; Mr. Brodie's practice is a modification of the latter. It is evident, that when the whole veins of the leg are in a state of morbid dilatation, little can be expected from any thing but the uniform pressure of a bandage. But when an irritable ulcer is connected with varicose vessels, or when there is a varix in one part of the leg, painful and liable to bleed, while the veins in the other parts are nearly sound, then Mr. Brodie has made an incision with a scalpel through the varix and skin over it, by which it was destroyed as completely as by caustic, and in a preferable manner. Latterly he has improved on this plan, which consists in making a small puncture in the skin over the varix, and then with a narrow sharp-pointed bistoury, slightly curved, and introduced between the skin and the varix, the division of the latter is effected, with however some pain, which soon subsides. Hæmorrhage ensues, but is easily restrained by pressure, which is useful in keeping the divided surfaces in contact, and promoting union by the first intention. The patient should be kept in bed for four or five days, and the bandage very gently removed, lest the new adhesions should be separated. Should adhesion not take place, granulations will soon complete the process. This operation has, in Mr. Brodie's hands, been always successful. When performed for varicose ulcer, the pain of the latter has ceased immediately, and the sore was healed in a few days. The operation has not been followed, in any case, by inflammation of the coats of the veins. In two or three instances, inflammation of the adipose and cellular membrane took place, producing local pain and some symptomatic fever; but these were easily subdued by the common antiphlogistic measures. This paper is characterized by utility.

Art. 14. *Last Illness of De Saussure.* By Dr. Odier, of Geneva.

It is not consistent with our plan to give more space to the diseases of the great than the humble. M. De Saussure laboured under a number of anomalous symptoms, particularly vertigo and pyrosis for some time before his death. These resisted every mode of treatment, and could not be explained till post mortem examination unravelled the plot. Effusion of water in the ventricles, and some morbid appearances in the membranes of the brain, accounted for the vertigo; while the pyrosis and vomiting were thought to arise from a peculiar conformation of the colon, which was much dilated, and pressed in a particular manner on the stomach. The dimensions of the thorax having been much encroached on, the lungs extended under the clavicles to some height along the neck.

To this long paper is appended some account of the last illness of Professor Ferguson. This gentleman was seized with hemiplegia at the age of 50. He was immediately bled and purged, and confined to low diet. By this treatment, the symptoms gradually abated; but the limbs on one side continued weak and flaccid. For the rest of his cure he trusted to time and a strict regimen; and he was not disappointed; for his paralytic symptoms having entirely disappeared, his health was so good in his 73d year as to excite admiration. The following is an outline of his regimen. 1st. Having an increased sensibilitys to the impression of cold, he guarded against these by additional cloathing on his legs and feet. He was also very attentive to his bed-clothes, varying them even in the same night, according to the state of the thermometer and his feelings. 2d. Goes to bed at ten o'clock, and sleeps with head high. 3d. Breakfast, tea and bread. 4th. Dinner, a bason of broth or bouillon of beef, mutton, or lamb, with a little barley or vegetables, eating with it bread, and often cabbage well boiled, finishing his dinner with potatoes or pudding. He drinks nothing but water. Tea and bread and butter two or three hours after dinner, and nothing afterwards. The only medicines he used were laxatives, which he took almost daily; generally aloes. This good state of health continued till nearly the period of his existence 1810, when he died of a fever.

We believe that the range of life might be very much prolonged by such a regime of abstinence as above; and that purgative medicines are, of all others, the best adapted to aid the good effects of regimen.

ART. 15. *Case of Chorea in an Adult.* By Mr. KINDER WOOD.

This occupies only 20 pages of the Medico-Chirurgical Transactions! Those who can afford time and patience for the perusal of a grave record of every motion in every muscle during a very long and daily St. Vitus's dance, performed to the tune of "protestant boys," by an active lass of 22 years, may consult the 7th volume of the Medico-Chirurgical Transactions. Dr. Watt's cases of periodical jactitation may now hide their diminished heads before this of Mrs. Whitworth. This lady kept time to the music of a fife and drum; and, what is not the case with every lady, the moment she made a *faux pas*, all her wild and extravagant motions disappeared. What is still more remarkable—"This woman, previously to her complaint, could never dance; and yet I saw her execute steps which could not be taught without difficulty." p. 248. In fact, St. Vitus's chapel never contained a lighter footed nymph at the annual feast; and here too the music was successful.

Art. 16. *A monstrous Fœtus.* By Mr. Munoir, of Geneva.

The Medico-Chirurgical *Transactions* have long been pregnant with *monstrous* productions. That the period of gestation may be near a close, and that the Medico-Chirurgical *Society* may soon be *delivered* of such unprofitable *burthens*, is the fervent prayer of the Medico-Chirurgical Journal!

Mr. Munoir very wisely concluded, that Switzerland was not the soil for perpetuating this shapeless monster by expensive and elegant engravings. No, no! Give, says he, John Bull something to stare at, and he never calculates the cost. The fœtus was, therefore, carefully packed up, and transmitted to London for the Society's Museum. And yet we are inclined to think, that "Mi lord Anglais, begins instinctively to grasp the strings of his purse in these times, and hesitate before he launches forth in unprofitable expences. The *ten guinea* members indeed care not how many useless plates may be appended to each volume; but we would hint to their High Mightinesses—" the President and Council," that the introduction of such *exotics* during the present coldness and barrenness of tne fields of medical literature, is at least injudicious, and that, it probably, will require all the artificial heat and genial influence of the Society to enable them to take root, much more to flourish.

Art. 17. *Cæsarian Operation.* By Kinder Wood, Esq.

A distorted pelvis, authorized here the Cæsarian Operation. A perpendicular incision six inches in length was made in the linea alba, one half above, the other below the umbilicus. By this, the peritonæum was exposed, slightly covered by the fibres of the linea alba. An opening being made at the lower part with a knife, the incision was completed upwards with the crooked scissars, when the uterus was brought fairly into view; and although no labour pains were present, the womb was rolling about. The knife being again resumed, an incision was made into the substance of the uterus, but not quite through. An opening being made in the lower part of this wound, the crooked scissars completed the opening upwards, as in the former instance. The right hand was passed in, and the fœtus extracted. It was dead. The placenta was next separated and brought away, when the uterus suddenly contracted; a rush of intestines presented at the wound. Six stitches were taken, and the wound secured with adhesive slips and bandage. Not more than two ounces of blood were lost. She died in ten hours.

*Dissection.* The edges of the wound looked like a fresh incision. The substance of the womb bore no marks of injury. The peritoneal coat was red and inflamed. The peritonæum itself was universally red and inflamed, as was the peritoneal coat of the intestines. A small quantity of bloody serum was effused within the cavity of the belly, and the external organs of generation were in a semi-gangrenous state. On this case we have only to remark, that we differ *toto cælo* from Mr. Wood on the employment of scissars in preference to the bistoury. Mr. W. must know, that the former invariably make a contused wound; and although the hæmorrhage be less, we know not whether even this be any advantage. In other respects, we think the paper very creditable to Mr. Wood, and very indicative of a strong mind and sound judgement. His manly exertions in this case deserved success; and we hope he will be more fortunate on a future occasion, should it be his fate to be called to a similar scene.

ART. 18. *On the Laceration of Muscular Fibres.* By JAMES WARDROP, Esq.

Mr. W. confines himself in this paper to the laceration of fibres in the external gastrocnemius muscle; generally the consequence of some untoward or sudden action of the muscle itself. The attention of the patient is first called to it by a sense of sharp pain in some part of the leg, most commonly at that part where the fibres become tendinous, accompanied by lameness. On examination, an inequality will be perceived at the pained part; a concavity being formed by the separation of the lacerated extremities of the muscular fibres, with tenderness to the touch, and, in a short time, swelling and tension of the whole calf. These symptoms continue with little abatement until by rest and proper means the inflammation have time to subside, and the lacerated parts to reunite. Every attempt at motion increases the pain and lameness. The lacerated extremities of the fibres should be placed as soon as possible in contact, and carefully retained in that situation until they adhere. A proper bandage must be applied, rest enjoined, and the antiphlogistic regimen prescribed.

Art. 19. *On a Change of Colour in the Skin from the internal Use of Nitrate of Silver.* [By Dr. Albers, of Bremen.

It seems now to be ascertained beyond a doubt, that a tinge of the skin, more or less permanent, has sometimes resulted from the internal use of lunar caustic.

A fat woman, 30 years of age, was seized with epileptic fits, and Dr. Albers prescribed the nitrate of silver in small doses. The woman continued the medicine (one-quarter of a grain daily) unknown to the Doctor, nearly three years and a half, without intermission. Toward the close of this period, a change in her complexion first became visible, particularly in the face .She was then pregnant. The tinge,at first bluish, grew gradually darker, till at last it became, and has since continued, almost black!

"Whenever the patient holds her arms in an erect posture, the blue colour is considerably lessened, and even disappears almost entirely."

Does Dr. A. mean that it disappears from the whole body or only from the arms? The blue colour is not always of the same intensity, varying several times a-day without any obvious reason. It is always deeper at the period of menstruation. The woman's blood is the same as that of a person in health. She is in other respects well; and has had no return, but once, of the epileptic fits. Various remedies have been tried, but all in vain.

Three other cases were communicated to Dr. Albers by Dr. Schleiden of Hambro. 1st. A lady, 35 years of age, and her sister 38 years of age, employed this remedy for epilepsy ten years ago. No cure was effected by it, but merely a less frequent return of the paroxyms. In both, the blue colour is visible at this very time, more particularly in those parts of the body exposed to the light, as the face, hands, neck, &c.

The third patient, a young man, 20 years of age, the son of an epileptic mother, was himself epileptic from birth. Various remedies being unsuccessfully employed, Dr. S. exhibited the nitrate of silver, five years ago. He continued it three months, rendering the fits much less frequent. Dr. S. does not say whether the skin was tinged or not in this case, though he surely must mean it.

*Additional Facts by Dr. Roget.*

A young lady, 25 years of age, was suddenly seized with epilepsy. After the fruitless exhibition of other remedies, Dr. R. directed the nitrate of silver in the form of pill, gradually increasing the dose from one to two grains three times a-day. At an interval of two months another fit occurred. The dose of the remedy was still farther increased, yet another attack took place in three weeks, but mild. The fits now occurred at irregular intervals of two or three weeks, and were, on the whole, becoming less violent, at length ceasing altogether. The dose of nitrate had now been increased to eighteen grains in the twenty-four hours. It was continued with intermissions, for some months, and then gradually left off. During the whole of the period that the patient was taking the medicine her health improved, and she got rid of a variety of nervous feelings to which she had been previously subject. Some time after leaving off the nitrate, she perceived her tongue and fauces of a darker colour than usual, which sometimes increased and sometimes diminished. In about eighteen months from the commencement of the medicine, and several months after its cessation, her complexion began to darken, and gradually increasing, acquired its maximum of intensity in the course of a year. It is liable to occasional variations without apparent cause. Of late years her health has been considerably deranged from the predominance of a nervous temperament, but no appearance of epilepsy.

The effect in question was noticed by Fourcroy, as appears by a passage in "*Medecine éclairée par les sciences physiques.*" "Ayant continué pendant plusieurs mois l'usage de ce remede [nitrique d'argent] sa peau s'altéra insensiblement, et elle devint enfin presqu'entierement noire." Tom. i. p. 342.

Dr. Butini, in an inaugural dissertation published at Geneva last year, (1815) relates three cases of dark hue in the skin following the use of nitrate of silver in epilepsy. Another case is detailed by Dr. Butini, as having occurred in the practice of Professor Delarive of Geneva. The epilepsy had affected the patient from the age of 12, and had continued with great obstinacy for four years. The nitras argenti was resorted to, gradually increasing the dose, half a grain every fortnight. By this plan the intervals between the paroxyms were lengthened. In consequence of a journey the medicine was intermitted, and the epilepsy increased. Then the remedy was resumed in doses of three grains daily, which were soon increased to six. Now the disease seemed to give way completely. But a blue tinge is beginning to be perceptible. In another recent case under Dr. Roget himself, the same effect is taking place.

These testimonies have now so multiplied, that it will become a point of delicate deliberation how far we can safely go with the remedy, especially among the female sex, and in the upper walks of life. Although 'tis not—

" The tincture of the skin that we admire;"

yet, few of our fair countrywomen would risk the acquisition of an Æthiopian complexion, even for an immunity from that direful fiend epilepsy.

We have now analysed every article in this volume, and we may conscientiously say, that we have not left a single useful fact, or ingenious speculation, unnoticed. The merit of such analyses cannot be unappreciated by the discerning reader.

Valeat quantum valere debet!

